# Morphologically Distinct *Escherichia coli* Bacteriophages Differ in Their Efficacy and Ability to Stimulate Cytokine Release *In Vitro*

**DOI:** 10.3389/fmicb.2016.00437

**Published:** 2016-03-31

**Authors:** Mohammadali Khan Mirzaei, Yeneneh Haileselassie, Marit Navis, Callum Cooper, Eva Sverremark-Ekström, Anders S. Nilsson

**Affiliations:** Department of Molecular Biosciences, The Wenner-Gren Institute, Stockholm UniversityStockholm, Sweden

**Keywords:** pharmacokinetics, phage therapy, cytokines, immune response, multi-resistant bacteria

## Abstract

Due to a global increase in the range and number of infections caused by multi-resistant bacteria, phage therapy is currently experiencing a resurgence of interest. However, there are a number of well-known concerns over the use of phages to treat bacterial infections. In order to address concerns over safety and the poorly understood pharmacokinetics of phages and their associated cocktails, immunological characterization is required. In the current investigation, the immunogenicity of four distinct phages (taken from the main families that comprise the *Caudovirales* order) and their interaction with donor derived peripheral blood mononuclear cells and immortalized cell lines (HT-29 and Caco-2 intestinal epithelial cells) were investigated using standard immunological techniques. When exposed to high phage concentrations (10^9^ PFU/well), cytokine driven inflammatory responses were induced from all cell types. Although phages appeared to inhibit the growth of intestinal epithelial cell lines, they also appear to be non-cytotoxic. Despite co-incubation with different cell types, phages maintained a high killing efficiency, reducing extended-spectrum beta-lactamase-producing *Escherichia coli* numbers by 1–4 log_10_ compared to untreated controls. When provided with a suitable bacterial host, phages were also able to actively reproduce in the presence of human cells resulting in an approximately 2 log_10_ increase in phage titer compared to the initial inoculum. Through an increased understanding of the complex pharmacokinetics of phages, it may be possible to address some of the safety concerns surrounding phage preparations prior to creating new therapeutic strategies.

## Introduction

Since their initial discovery, bacteriophages have been used as antibacterial agents in a number of specialist centers in Eastern Europe. However, their initial clinical successes were later overshadowed by the efficiency and increased use of antibiotics in Western medicine. Nonetheless, the global spread of multi-drug resistant bacteria has revitalized research into phage biology and its applications, as new strategies are sought to combat antibiotic resistant bacterial infections (Kutter et al., 2010; Nilsson, 2014). Although phage therapy possesses a number of advantages over antibiotics, including the relative ease of isolation of new phages compared to finding new antibiotics as well as ability to self-replicate, concerns over efficacy and safety have also hindered their adoption in the West for use in humans (Abedon, 2010, 2011).

In order to successfully combat a bacterial infection, phages must first reach the site of infection in sufficiently high concentration to be able to reproduce to an extent that eventually all or almost all bacteria will become infected. This requires not only high initial doses of phages but also that the phages need to persist in the presence of, or where possible evade, the neutralizing effect of the patients immune system. Although initially believed to be non-immunogenic and non-toxigenic (Adams, 1959) recent studies have shown that high concentrations of phage can induce pro-inflammatory responses, while long term exposure to phages could induce an antibody response in which the spleen plays a key role in phage clearance, which has been demonstrated to be the case for phages T4 and T7 (Dabrowska et al., 2014; Majewska et al., 2015; Hodyra-Stefaniak et al., 2015). Among the safety concerns surrounding the use of phages to combat bacterial infections is also the potential to induce toxic shock caused by the release of lipopolysaccharide (LPS) as a result of bacterial lysis. The injection of low doses of such endotoxins to the human body has been shown to trigger inflammatory responses, and the endotoxin content of pharmaceutical preparations is therefore tightly controlled during manufacture (Merabishvili et al., 2009; Neyen and Lemaitre, 2015). In order to be of clinical use, phage preparations would also have to conform to varying regulatory standards that are contingent on route of administration, prior to approval for use.

In the current study, the cytokine response of human intestinal epithelial cells (IEC) and immune cells to four distinct phages was evaluated in addition to the mitogenic or cytotoxic effects of the phages on these cells.

## Materials and Methods

All experiments in the current study were performed in triplicate unless otherwise stated in the text.

### Bacterial and Bacteriophage Culture

*Escherichia coli* strains (ECOR10, ECOR32, ECOR57, and ECOR63) were obtained from the *E. coli* reference collection (ECOR; Ochman and Selander, 1984), and were routinely cultured in lysogeny broth (LB) at 37°C with shaking at 120 RPM.

*Escherichia coli* phages SU10 (*Podoviridae*), SU32 (*Siphoviridae*), SU57 (*Siphoviridae*), and SU63 (*Myoviridae*) were isolated from the Käppala Wastewater treatment works, Lidingö, Stockholm county, Sweden, and have been characterized elsewhere (Khan Mirzaei et al., 2014; Khan Mirzaei and Nilsson, 2015). Phages were routinely cultured in LB with appropriate hosts (ECOR10, ECOR32, ECOR57, and ECOR63) prior to purification in PEG-8000 and CsCl gradient centrifugation as previously described (Villafane, 2009). Phages were enumerated using the agar overlay method as previously described (Khan Mirzaei and Nilsson, 2015). Extended-spectrum beta-lactamase producing *E. coli* strains, here denoted ESBL-32 (strain 07RAFM-ECO-32) and ESBL-198 (strain 07RAFM-ECO-198), were grown in RPMI-1640 complete medium (Invitrogen, Sweden) supplemented with 10%(v/v) heat inactivated fetal calf serum (FCS; Invitrogen), 1%(v/v), penicillin–streptomycin (PEST; Thermo Scientific, Logan, UT, USA), 2%(v/v) L-glutamine and 4%(v/v) HEPES (HyClone Laboratories, Inc, South Logan, UT, USA). ESBL-198 was used for the infection of SU32, SU57, and SU63 while ESBL-32 was used due to the limited host range of SU10.

### Preparation of Bacterial Debris

Bacterial debris was prepared from a 1.5 L overnight culture, centrifuged at 11000 × g for 20 min and re-suspended in 20 mL of phosphate buffered saline (PBS) then sonicated with 80% power for 15 min in a VCX130 Vibra-Cell (Sonics & Materials Inc., Newtown, CT, USA) to break up bacterial cells. Bacterial debris samples were subjected to CsCl purification as previously described (Villafane, 2009). In order to confirm the efficacy of debris removal, samples were taken from the same point in the ultracentrifuge tube as phage stocks.

### Stimulation of HT-29 and Caco-2 Cell Lines by Purified Bacteriophages

HT-29 (HTB-38) and Caco-2 (HTB-37) were cultured at 37°C in a 5% CO_2_ atmosphere in McCoy’s 5A (modified) medium (ATCC or HyClone Laboratories Inc.) and in Dulbecco’s modified Eagle’s medium (DMEM; HyClone Laboratories, Inc.), respectively. Both media were supplemented with 10%(v/v) FCS and 1%(v/v) PEST. To maintain exponential growth phase, cells were treated with trypsin-EDTA (Invitrogen) and sub-cultured before reaching confluence (Haileselassie et al., 2013).

In order to provide stimulation, HT-29 and Caco-2 cells were trypsinated, washed in fresh medium and seeded at a final concentration of 2 × 10^5^ cells/well for cytokine analysis in 48 well plates (Costar, Cambridge, UK) and 5 × 10^4^ cells/well in flat-bottomed 96-well plates (Sarstedt Inc., Newton, NC, USA) and grown over night at 37°C in a 5% CO_2_ atmosphere. Following incubation, both Caco-2 and HT-29 cells were stimulated as follows: (1) addition of phages alone (10^9^ PFU/well), (2) addition of bacteria alone (10^5^ CFU/well), (3) simultaneous addition of phages and the host bacteria (10^9^ PFU/well and 10^5^ CFU/well), and (4) complete media only. Cells were incubated for 8 h for cytokine analysis and 72 h for 3-(4,5-Dimethylthiazol-2-yl)-2,5-Diphenyltetrazolium Bromide (MTT) analysis at 37°C in 5% CO_2_. Supernatants from IEC stimulations were collected and frozen at -20°C until cytokine analyses and the cells were subjected to MTT analysis.

### Human Cytokine Array

To profile the response of stimulated HT-29 epithelial cells, 38 cytokines were simultaneously analyzed using a Human cytokine array kit (Cat no. ARY005, R&D Systems, Abingdon, UK) according to the manufacturer’s instructions. Qualitative analysis was performed by comparing the density of the detected spots to that of the reference spots included in the assay.

### Stimulation of PBMCs by Purified Bacteriophages

#### Ethical Permission

Peripheral blood mononuclear cells (PBMCs) from healthy blood donors were obtained. The samples were treated according to the by-law of the ethical permission granted by the Karolinska Institute, Stockholm, Sweden (Permit no. 2014/2052-32).

#### PBMC Preparation

PBMC were isolated by Ficoll–Hypaque (GE Healthcare Bio-Sciences AB, Uppsala, Sweden) gradient centrifugation and then cyro-preserved in liquid nitrogen. Prior to use, cells were thawed and washed three times in pre-warmed RPMI-1640 complete medium (Invitrogen, Sweden) supplemented with 10%(v/v) FCS, 1%(v/v) PEST, 1%(v/v) L-glutamine, and 2%(v/v) HEPES (HyClone Laboratories, Inc.) and checked for viability. PBMC (1 × 10^6^ cells/mL) were then seeded into flat-bottomed 96-well plates (Sarstedt Inc.) and co-cultured with three different concentrations of four phages (10^9^, 10^7^, and 10^5^ PFU/well) for 24 h for cytokine analysis and a single concentration of 10^9^ PFU/well for 96 h for MTT assay at 37°C in a 5% CO_2_ atmosphere. For cytokine analysis, negative (complete media only) and positive (50 ng/mL LPS) controls were also performed. For MTT assays, cells treated with water and dimethyl sulfoxide (DMSO) acted as positive controls for cell toxicity, complete medium was used as a negative control and a-CD3/a-CD28 beads (Invitrogen) acted as a proliferation control. Supernatants from PBMC stimulations were collected and frozen at -20°C until analyzed for cytokine content and the cells were used for MTT assay.

#### ELISA for Cytokine Stimulation of PBMC

ELISA was performed on purified phage preparations of different concentrations (10^9^, 10^7^, and 10^5^ PFU/well) using commercially available kits IL-2, IL-4, IL-6, IL-10, IL-17, IFN-γ, and TNF-α (Mabtech AB, Stockholm, Sweden) according to the manufacturer’s instructions. Optical density (OD) was determined at 405 nm on a micro-plate reader (Molecular Devices Corp, Sunnyvale, CA, USA). Data were analyzed in the SoftMax Pro 5.2 rev C software package (Molecular Devices Corp, Sunnyvale, CA, USA).

### Cell Proliferation and Toxicity Assay

Post-stimulation, 100 μL/well of their respective complete medium containing 9.1% 12 mM MTT solution (Vybrant^®^ MTT Cell Proliferation Assay Kit (Thermo Fisher Scientific, Sweden) was added into the PBMC and IEC (Caco-2 and HT-29) culture wells and the plates were incubated at 37°C for 3 h. MTT solution containing complete media was also added in an empty well as blank control. The supernatant was then removed, and the purple formazan crystals formed were solubilized with 50 μL/well of DMSO (Sigma-Aldrich, MO, USA). The absorbance was determined using an automatic microplate spectrophotometer at 540 nm. The OD values for each stimulus were adjusted for the contribution of the blank control (Peng et al., 2007).

### Endotoxin Quantitation

Purified phage preparations and bacterial debris were checked once for endotoxin content using a Pierce Limulus amebocyte lysate (LAL) Chromogenic Endotoxin Quantitation Kit (Thermo Fisher, Sweden) according to the manufacturer’s instructions.

### Statistical Analysis

A one way ANOVA was used for the analysis of normally distributed whole data sets, 2-tailed *t*-tests for pairwise comparisons within normalized data sets, Kruskal–Wallis tests for non-normally distributed whole data sets or Mann–Whitney for pairwise comparisons between non-normalized data. Statistical analysis was performed in the *R* Statistical software package (R Core Team, 2015). Individual statistical tests used are identified in the figure legends or text where necessary.

## Results

### Phages Maintain Lytic Activity in the Presence of Human Epithelial Cells

**FIGURE 1:**
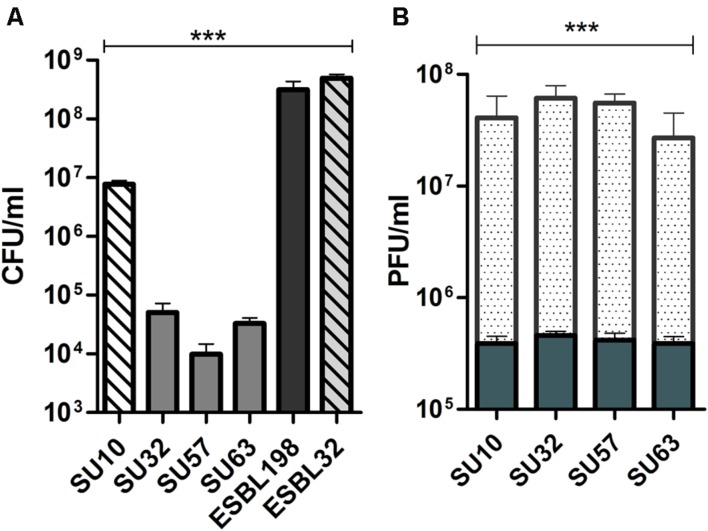
**Bactericidal activity and proliferation of phages in the presence of human epithelial cells. (A)** Bactericidal efficacy of phages in the presence of epithelial cells. Efficacy is compared to bacteria only controls. Solid bars are ESBL198. Hatched bars are ESBL32. **(B)** Active replication of phages in the presence of host and epithelial cells; solid bars are phages and epithelial cells only, dotted bars are phages in the presence of host and epithelial cells. Reproduction in the presence of host is compared to phages exposed to epithelial cells. Results from HT-29 (*n* = 3) and Caco-2 (*n* = 3) cells were combined and data are presented as mean ± SD ^∗∗∗^*P* < 0.0001 by one way ANOVA.

When incubated with epithelial cells and hosts, phages were able to significantly reduce the bacterial content over a period of 8 h (**Figure 1A**; *P* < 0.0001 by ANOVA). This reduction in bacterial content was shown to be the result of productive phage replication by an increase in phage titer over the same period (**Figure 1B**; *P* < 0.0001 by ANOVA). It should also be noted that all phages, were reduced in number following 8 h incubation with epithelial cells when compared to the initial inoculum (from 5 × 10^5^ to the lowest 3 × 10^5^; *P* < 0.05 by two tailed *t*-test). For SU32 this reduction was smaller (from 5 × 10^5^ to the lowest 4 × 10^5^; *P* > 0.05 by two tailed *t*-test). No significant impact on the efficacy and stability of phages was observed between the two cell lines.

### Phages Activate Pro-inflammatory Cytokines in HT-29 Cells

When phages were incubated with HT-29 cells in the absence of a suitable host, cytokines IL-8, CXCL-1/GROα and Macrophage migration inhibitory factor (MIF) were induced (**Table 1**). In the presence of a bacterial host (ESBL-198), a more intense pattern of cytokine activation was observed for phages SU32, SU57, and SU63. An additional cytokine, IL-1ra/IL-1F3 was activated in the presence of ESBL-198 only.

**Table 1:** Cytokine release from HT-29 cells when exposed to four structurally distinct phages.

Cytokine	Bacteriophage				Bacteria and phage				Controls		
	SU10	SU32	SU57	SU63	ESBL-198			ESBL-32	ESBL-198	ESBL-32	Medium
					SU32	SU57	SU63	SU10			
IL-8	++	+	+	+	++	+++	+++	++	+++	+++	-
IL-18/IL-1F4	-	-	-	-	-	-	-	++	-	++	-
CXCL1/ GROα	++	+	+	+	++	++	+++	++	++	++	-
IL-1ra/IL-1F3	-	-	-	-	-	-	-	+++	++	+++	-
MIF	+	++	++	++	++	++	++	+++	+++	+++	++

*^+++^Pixel intensity ≥ reference spot.*

*^++^Pixel intensity < reference spot.*

*^+^Pixel intensity < than reference and test (bacteria and phage) spots.*

*^-^No response detected.*

Due to the host range of SU10, a second ESBL containing *E. coli* (ESBL-32) was chosen for comparative analysis. This combination of phage and host induced an additional pair of cytokines, IL-18/IL-1F4 and IL-1ra/IL-1F3 in the presence of HT-29 cells (**Table 1**). These additional cytokines were also present in the ESBL-32 control.

### Inflammatory and Anti-inflammatory Immune Response Varies between Phages

**FIGURE 2:**
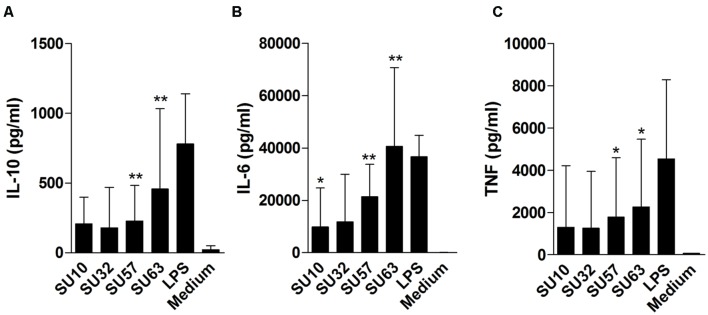
**Cytokine release by peripheral blood mononuclear cells (PBMCs) following exposure to high concentrations (10^9^ PFU/well) of four different purified bacteriophages. (A)** IL-10 release. **(B)** IL-6 release. **(C)** TNF-α release. Data are the mean of two replicates taken from six donors ± SD. ^∗∗^*P* < 0.001; ^∗^*P* < 0.05 by Mann–Whitney. Cytokine induction by phages is compared to medium.

When assessed at the highest phage concentration (10^9^ PFU/well) all samples were found to induce IL-6, IL-10, and TNF-α (**Figure 2**) at varying levels depending on the phage/cytokine combination. When compared to the medium (negative) control, IL-6 was significantly induced by all phages (*P* < 0.002 by Mann–Whitney) with the exception of SU32. Both IL-10 and TNF-α were significantly induced by SU57 and SU63 (*P* < 0.05 by Mann–Whitney) but not by SU10 or SU32 when compared to the negative control. When compared to the positive (LPS) control, all phages were significantly less immunostimulatory (*P* < 0.05 by Mann–Whitney) with the exception of SU63. TNF-α induction levels for SU57 were insignificant when compared to LPS. When comparing the ratio of TNF-α to IL-10, phage treated samples were higher than the medium control (*P* > 0.05 by Mann–Whitney). At lower phage concentrations (10^7^ and 10^5^ PFU/well) no significant induction of cytokines were observed (data not shown). IFN-γ was induced by all four phages but only in PBMCs obtained from two donors. The levels of IL-2, IL-4, and *IL-17* were below the limit of detection for all conditions tested. In addition, no response to the purified bacterial debris when incubated with PBMC was observed (data not shown).

### Phages Are Non-toxic to Human Cells

**FIGURE 3:**
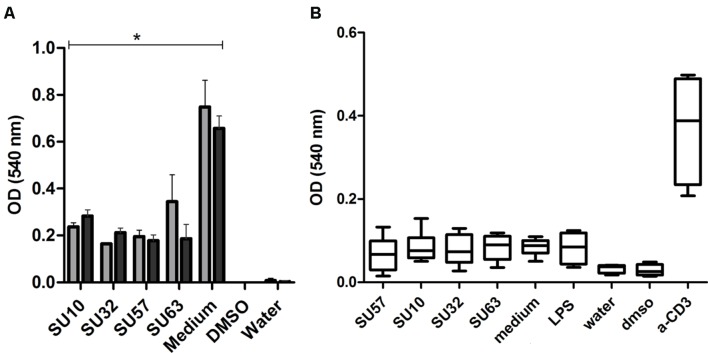
**Cell cytotoxicity and inhibition of proliferation by exposure to phages. (A)** Phage exposure to intestinal epithelial cells; HT-29 (*n* = 4) 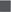 and Caco-2 (*n* = 4) 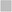. Data are presented as the mean ± SD. ^∗^*P* < 0.05 by two tailed *t*-test compared to the medium control. **(B)** Phage exposure of PBMCs. Data are from two replicates from four donors.

In a long term assessment of the cytotoxic effects of individual phages on IECs and PBMCs, phages at a concentration of 10^9^ PFU/well were not lethal to the HT-29 and Caco-2 cells after 96 h of incubation (*P* < 0.05 by two tailed *t*-test) and no significant differences between the cell lines were observed (**Figure 3**). Phage stimulated IECs produced a significantly (*P* < 0.05 by two tailed *t*-test) higher OD response compared to the negative control. Phages were unable to induce a significant cytotoxic effect on PBMCs.

### LAL Analysis of Phage Preparations May Not Be Suitable for Quality Control Purposes

Phage preparations at the highest concentrations (10^9^ PFU/mL) showed marked differences in endotoxin content compared to the bacterial debris control.

## Discussion

Phage therapy has enjoyed renewed interest for the treatment of multi-drug resistant bacteria due to increasing global levels of resistance. Despite this renewed interest, a number of concerns still exist over their safety and efficacy, which require substantial investigation. Depending on the route of administration, phages would require a level of persistence in the presence of, or the ability to evade, innate and adaptive immune components in order to reach a target site and produce a therapeutic effect (Abedon, 2011; Chan and Abedon, 2012). In the current study, all phages were shown to maintain killing efficacy and replicative ability in the presence of IECs and a suitable bacterial host. However, individual phages varied in their ability to elicit an immunogenic response with SU57 (Siphoviridae) and SU63 (Myoviridae) being the most immunogenic and SU32 (Siphoviridae) the least and suggests that phage immunogenicity cannot be generalized between families (e.g., SU57, SU63) or within the same family (e.g., SU32 and SU57). Despite the temptation to generalize the immunogenic properties of phages, recent studies have demonstrated that different proteins within the T4 phage possess different levels of immunogenicity (Dabrowska et al., 2014; Majewska et al., 2015). In order to ascertain the differences in immunogenicity between phage families (e.g., Myoviridae, Siphoviridae, or Podoviridae) or individual phages (e.g., SU63 and SU32 in the current work), comparisons between protein content and tertiary structures would be required.

### Impact on Phage Efficacy and Viability When Exposed to Human Epithelial Cells

In order to be a viable adjuvant or alternative to conventional antibiotic therapy, whole phage therapeutics requires the establishment of a productive infection lifecycle. Following administration, this would require the migration of phages to the site of bacterial infection and the ability to persist in the presence of, or evade, the immune system while maintaining an infectious dose (Abedon and Thomas-Abedon, 2010; Abedon, 2011; Bull and Gill, 2014). ESBL-32 was chosen as a specific host for SU10 for the current investigation in order to produce comparable levels of activity between the four phages (Khan Mirzaei and Nilsson, 2015). Despite the maintenance of high killing efficacy and reproductive ability in the presence of IECs and suitable bacterial hosts (**Figure 1**), the number of phages reduced with a different level in the absence of bacterial hosts compared to the initial inoculum which could be due to difference in their stability under the experimental conditions. However, this low level of clearance would not explain the disparity between the activities of different phages, i.e., approximately 2–3 log_10_ difference between SU10 and the other phages and may result from the unique infection dynamics of each phage (Bull et al., 2014). Although not considered in this study, the *in situ* environmental conditions (e.g., gut pH) may potentially have a detrimental effect on the stability and efficacy of the phages.

### Cytokine Release and Immune Stimulation Following Phage Exposure

The current study shows the varying degrees of immunogenicity exhibited by four distinct phages of the Caudovirales order when stimulating human PBMCs and IECs. IL-18 activation is of current interest as a vaccine adjuvant due to the ability to activate T-cells in the presence of different bacteria and cell types (Miettinen et al., 1998; Sugimoto et al., 2004). It is generally believed that phages are safe for human use, however, the lack of appropriate clinical trials and a lack of regulatory approval have restricted routine clinical use to specialist centers around the world (Skurnik and Strauch, 2006; Sarker et al., 2012). However, the current investigation suggests that both SU57 and SU63 at high concentrations (10^9^ PFU/well) can significantly induce IL-6 and TNF-α from PBMCs compared to the negative (medium) control. SU57 and SU63 were also able to significantly induce the anti-inflammatory cytokine IL-10 compared to the negative (medium) control. In addition to this, SU10 also activates IL-6 release with a significant level from PBMCs compared to the negative (medium) control. Despite a lack of toxicity in both HT-29 and Caco-2 cells, phage exposure inhibited cell proliferation via an unknown mechanism although there is some suggestion that the interaction between phages and epithelial cells may result in the rapid release of reactive oxygen species (Gorski et al., 2012).

### Endotoxin Determination in Phage Preparations

In order to satisfy regulatory requirements for non-topical clinical application, particularly intravenous injection, it is necessary to quantify endotoxin levels in the final product (Merabishvili et al., 2009). This is routinely accomplished with the “gold standard” method of an LAL assay. However, a number of studies suggest that the use of LAL assays results in ambiguous results when applied on phages (Merabishvili et al., 2009; Cooper et al., 2014) and was again highlighted in the current study. This discrepancy between LAL results and immune response studies may be the result of phages/LAL cross reactivity. Although supplemental methods, including *in vivo* assessments exist, these methods may not provide the required level of sensitivity necessary for pharmaceutical quality control and also raise ethical concerns over routine animal usage. An ELISA assay to profile immune responses to formulations may provide a sensitive alternative to LAL assessment and address ethical concerns over routine animal usage as has been suggested by Hodyra-Stefaniak et al. (2015) and should be the focus of future work.

## Conclusion

The level of immunogenicity varies between the phages tested with SU57 and SU63 stimulating a greater release of IL-10, IL-6, and TNF-α compared to SU10 and SU32 in PBMCs. Despite this immunostimulatory activity, all phages were able to maintain high levels of lytic activity and establish reproductive cycles in the presence of suitable bacterial hosts and IECs. This may suggest that should sufficient numbers of phages reach a site of infection then a therapeutic effect may arise. All phages halted the growth rate of IECs compared to the control medium. The current investigation has highlighted the importance of immunological studies for the development of phages for use in human therapy and also that cell line studies may provide an additional method for endotoxin detection and quantification.

## Author Contributions

Conceived and designed the experiments: MKM, YH, ES-E, AN. Performed the experiments: MKM, YH, MN, CC. Analyzed the data: MKM, YH, CC, AN, ES-E. Wrote the manuscript: MKM, CC, YH, AN, ES-E.

## Conflict of Interest Statement

The authors declare that the research was conducted in the absence of any commercial or financial relationships that could be construed as a potential conflict of interest.
