# Corrigendum: Morphologically Distinct *Escherichia coli* Bacteriophages Differ in Their Efficacy and Ability to Stimulate Cytokine Release *In Vitro*

**DOI:** 10.3389/fmicb.2016.02145

**Published:** 2017-01-04

**Authors:** Mohammadali Khan Mirzaei, Yeneneh Haileselassie, Marit Navis, Callum Cooper, Eva Sverremark-Ekström, Anders S. Nilsson

**Affiliations:** Department of Molecular Biosciences, The Wenner-Gren Institute, Stockholm UniversityStockholm, Sweden

**Keywords:** pharmacokinetics, phage therapy, cytokines, immune response, multi-resistant bacteria

In the original article, it was suggested that bacterial debris controls exhibited no cytokine response when incubated with PBMC. However, a subsequent data audit and additional statistical analysis has revealed that a number of the bacterial debris controls exhibited a positive cytokine response whereas others not, resulting in an inflated mean particularly for the TNF response (Supplementary Table 1). These means were not significantly different to the response generated by the purified phages. This does not impact on the data presented or the statistical analysis that has been performed as part of Figure 2 (analysis was compared to commercial LPS or medium only) or any of the other analysis performed as part of the manuscript. However, it does mean that the bacterial debris controls are not suitable for showing the efficacy of the phage purification process and as such a component of the cytokine response generated may be due to remaining bacterial debris as suggested by Dufour et al. (2016).

Therefore, the sentence “In addition, no response to the purified bacterial debris when incubated with PBMC was observed (data not shown)” (page 4, end of second paragraph) is inaccurate and should consequently not be considered when evaluating the effect of phage preparations on human cell lines as described in our article.

In addition, there is an error in the figure legend of Figure 3A. The colors used to differentiate between the HT-29 and Caco-2 cells have become inverted.

The authors apologize for these two mistakes. While the first does not impact the conclusions from the statistical analyses that have been performed, as the comparisons of immune response of phage preparations by PBMCs were made against a standardized LPS control and not against the purified bacterial debris controls, it does support the suggestion made by Dufour et al. (2016) that at least some component of the observed cytokine response generated by the phage preparations may be due to residual contaminants.

## Conflict of interest statement

The authors declare that the research was conducted in the absence of any commercial or financial relationships that could be construed as a potential conflict of interest.
